# The Effect of Sign Language Structure on Complex Word Reading in Chinese Deaf Adolescents

**DOI:** 10.1371/journal.pone.0120943

**Published:** 2015-03-23

**Authors:** Aitao Lu, Yanping Yu, Jiaxin Niu, John X. Zhang

**Affiliations:** 1 Center for Studies of Psychological Application & School of Psychology, South China Normal University, Guangzhou, China; 2 Guangdong Key Laboratory of Mental Health and Cognitive Science, Guangzhou, China; 3 Guangdong Center of Mental Assistance and Contingency Technique for Emergency, Guangzhou, China; 4 Department of Psychology, Fudan University, Shanghai, China; The University of Science and Technology of China, CHINA

## Abstract

The present study was carried out to investigate whether sign language structure plays a role in the processing of complex words (i.e., derivational and compound words), in particular, the delay of complex word reading in deaf adolescents. Chinese deaf adolescents were found to respond faster to derivational words than to compound words for one-sign-structure words, but showed comparable performance for two-sign-structure words. For both derivational and compound words, response latencies to one-sign-structure words were shorter than to two-sign-structure words. These results provide strong evidence that the structure of sign language affects written word processing in Chinese. Additionally, differences between derivational and compound words in the one-sign-structure condition indicate that Chinese deaf adolescents acquire print morphological awareness. The results also showed that delayed word reading was found in derivational words with two signs (DW-2), compound words with one sign (CW-1), and compound words with two signs (CW-2), but not in derivational words with one sign (DW-1), with the delay being maximum in DW-2, medium in CW-2, and minimum in CW-1, suggesting that the structure of sign language has an impact on the delayed processing of Chinese written words in deaf adolescents. These results provide insight into the mechanisms about how sign language structure affects written word processing and its delayed processing relative to their hearing peers of the same age.

## Introduction

Empirical evidence indicates that deaf children generally show lower levels of reading comprehension than their hearing peers [1–2]. For example, their vocabulary size is smaller [3], and their vocabulary knowledge is less deep [1]. Such delay in reading comprehension in deaf children is argued to be primarily due to their difficulty in using phonology in written word reading [4–5]. Recent studies showed that poor print morphological awareness in deaf children also contributes to their reading comprehension difficulties due to their lack of familiarity with the meaning of the bound morphemes [6]. It remains however unclear whether the problem in print morphological processing in deaf children is due to their acquisition of sign language. The present study extends previous work on poor print morphological awareness in deaf children by focusing on the role of sign language structure.

Considerable evidence has accumulated suggesting that the bilingual language system is a dynamic system that can interactively operate in different language activation states. For example, bilingual lexical access has been repeatedly demonstrated to be non-selective at early stages of processing [7–8]. The facilitative effects obtained for cognates (e.g., tomaat-tomato) suggest that the common semantic representation between the two readings of a cognate exerts strong feedback activation to their orthographic and phonological representations [9–10]. Such co-activation is further supported by experimental evidence that highly proficient and less proficient bilinguals activate all of their languages in parallel even when only one language is used or needed [11–12].

Recently, several studies have documented that the bimodal bilinguals who know both a sign language and a spoken language can also produce lexical items from both languages at the same time [13–14], suggesting that the parallel activation of more than one language is not modality-specific. Sign language is very different from written and spoken language in that they are composed of signs corresponding to locations and movements along with facial expressions. Clearly there is no overlap in phonological awareness, morphological awareness, and orthographic knowledge between a spoken language and a sign language. However, is it possible that the activation of sign language has a bottom-up effect on written word reading, such as the role of sign language structure in print morphological processing? Morford et al. [15] was to our knowledge the only one that focused on investigating the effect of sign language (i.e., phonology) on print processing in unimodal bilinguals. Based on the results that deaf bilinguals with American Sign Language as the first language and English as the second language made faster semantic relatedness judgment on word pairs that were semantically related and had similar form of the ASL translation, but slower judgment on word pairs that were semantically unrelated but had similar form of the ASL translation, they aruged the automatic activation of sign language in written word processing. Different from Morford et al. (2011)’s study, the current study tried to examine whether the morphology of sign language (i.e., the structure of sign language) had an effect on print reading. As deaf children have difficulties in using phonology in written word reading, they may rely more on morphology to help identify written words and their meaning [16–17]. The reliance on visual morphology may provide a chance for us to see whether the structure of sign language affects their equivalent written word processing.

So far, several studies have investigated print morphological awareness in deaf readers and found that they could decompose words into morphemes and assign meaning to morphemes when reading written words in alphabetic languages. For example, Gaustad, Kelly, Payne, and Lylak [18] showed that deaf college students and hearing middle school students appeared to have approximately the same morphological knowledge and word segmentation skills. In a follow-up study by Gaustad and Kelly [19], the comparison of specific morpheme knowledge and word segmentation showed clear differences in the morphographic skills between hearing middle school readers and deaf college students, even though they were matched and appeared to read at the same grade levels. More recently, van Hoogmoed, Knoors, Schreuder, and Verhoeven [6] found that deaf children were delayed in reading both derivational words and compounds as compared to hearing children, but could successfully perform a lexical decision task.

The existing research converges by showing that deaf people are able to split words into morphemes and understand the meaning of alphabetic derivational and inflectional words. The question that follows is whether Chinese deaf readers also show print morphological awareness in Chinese written word processing. Chinese language possesses linguistic properties distinct from those of most widely studied European languages. As vocabulary development is cumulative, the differences between alphabetic language and logographic language are compounded over time [20]. Thus, it is expected that the ability to use print morphology and print morphological awareness in deaf people may well vary across languages, such as alphabetic English and logographic Chinese.

It is generally accepted that Chinese words are more closely mapped to meaning than to phonology compared with alphabetic languages [21]. A Chinese character is simultaneously a visual whole, a syllabic unit, and a morpheme, which is different from the units of writing in alphabetic scripts. This script-sound-meaning convergence of the Chinese character can facilitate the understanding and retrieval of the meaning of multi-character words as the component morphemes of multi-character Chinese words provide meaningful cues [22–23]. Thus, logographic Chinese is considered to have ‘deep’ orthography. Regardless of importance of characters in the Chinese language, many investigators have demonstrated the presence of lexical representations of Chinese words [24]. However, no one has considered the possible processing differences between different types of words (i.e., derivational words and compounds) in Chinese, or made any effort to investigate the delayed processing of these two types of words in Chinese deaf people.

Most previous research on morphological processing of Chinese has focused on Chinese compound words [25–26], which may be due to the fact that 70% of modern Chinese words are compounds [21]. Derivational words involve the addition of a morpheme in the form of an affix, such as -ness, un- and -tion in English and “-后” (-*hou*), “-们” (-*men*), “-子” (-*zi*) in Chinese to form a new word, e.g., happiness from happy, or “我们” (*women*, we) from “我” (*wo*, I). Compound words are made when two words are joined to form a new word, such as “elsewhere” and “grandmother” in English and “果园” (fruit +garden, meaning orchard) and “火车” (fire + car, meaning train) in Chinese. However, unlike derivational morphology in alphabetic language, Chinese derivational words have a specific appearance in word formation [27]. Most Chinese derived words (83.0%) have an internal structure with a suffix part. That is, derived words can be easily recognized by the arrangement of word components in Chinese, while compound words depend on the internal syntactic rules [27]. Thus, it is expected that the processing of Chinese derivational morphology may differ from that of Chinese compound words.

To summarize, previous studies have shown that deaf adolescents can acquire print morphological knowledge in alphabetic languages with their reading comprehension being worse than that of their hearing peers. However, it is unclear whether Chinese deaf adolescents also demonstrate print morphological awareness in Chinese written word reading. In addition, most previous research has focused only on morphological processing of Chinese compound words (CW), but not studied the possible different processing mechanisms between derivational words (DW) and compound words. More importantly, to our knowledge, there has been no study that investigates the impact of sign language structure (i.e., morphology) on complex word reading, nor on the delayed processing of complex word reading in deaf adolescents. Therefore, the present study was primarily intended to address the following four issues: whether sign language structure plays a role in the processing of complex words in deaf adolescents, whether Chinese deaf adolescents show print morphological awareness, whether the delay in complex word processing occurs in Chinese deaf adolescents, and if so, whether sign language structure has any effect on such delays.

We selected half of the complex words to be words formed of one sign and the other half formed of two signs. For deaf adolescents, it is assumed that the sequential presentation of two signs will make it difficult to access the lexical representation of two-sign words in sign language lexicon as one has to spend time integrating the two sequential signs first to understand the word, while it will be more efficient to access the meaning of one-sign words as no such integration would be needed. Thus, the contrast between one-sign and two-sign words will shed light on the effect of sign language structure on written word processing.

Moreover, according to findings that Chinese derivational words and compound words are different from each other in morphological structure with derivational words easier to be recognized and understood by the arrangement of word components while compound words depending heavily on the internal syntactic rules and comprehension through the integration of two base words, we postulated that the effect of sign language structure on Chinese word reading varies as a function of word type. Specifically, the meaning of DW-2 words (derivational word-2 signs) could be accessed more easily in written Chinese without additional integration of component characters, though needing additional processing to combine the two signs. The two-sign structure would have an inhibitory effect. For CW-2 words, their meaning can be accessed based on the integration of two component characters or the integration of two signs, which could occur at the same time. The inhibition of two-sign structure on CW-2 would be weaker than that in DW-2 words. For CW-1 words, their meaning is accessed based on the integration of two component characters and the one-sign structure would facilitate this meaning access. For DW-1 words, their meaning can be much easily accessed in both written Chinese and sign language without additional integration of characters or signs. Thus, the facilitation of one-sign structure of sign language on DW-1 would be weaker than that in CW-1.

Additionally, the present study was also intended to reveal the specific delay of word comprehension in Chinese deaf adolescents compared to their hearing peers. It is predicted that the delay in complex word reading in Chinese deaf adolescents may vary as a function of the word type (i.e., derivational words and compound words) and sign language structure (i.e., one-sign words and two-sign words), which will to some extent shed light on whether the structure of sign language has any effect on the delayed development of Chinese morphological awareness. Because the unique contribution of print morphological awareness to language reading appears to increase with age as reading skill develops [28], the present study recruited deaf adolescents aged 15-years-old or older to increase the likelihood of detecting the possible effect of sign language structure on word reading.

## Method

### Participants

#### Deaf adolescents

Eighteen deaf students (12 males, mean age = 17.8 ± 1.53 years) from a school for special education in Guangzhou participated in this experiment for monetary compensation. All were right-handed native Chinese Sign Language (CSL) signers and reported using CSL as their primary and preferred language. Handedness was assessed by the Edinburgh Handedness Survey [29]. All had normal or corrected-to-normal vision, and normal IQ as measured by an intelligence screening test for Chinese primary and secondary students, developed by Jin et al. [30]. They were all born deaf or became deaf before the age of two. None had any additional handicaps or cochlear implantation. According to their teachers, all of them had good Mandarin Chinese vocabulary skill.

#### Hearing adolescents

These participants comprised twenty native Mandarin Chinese hearing speakers (10 males, mean age = 17.5 ± 1.28 years) from a hearing school in Guangzhou. All were right-handed as assessed by Edinburgh Handedness Survey, and had normal or corrected-to-normal vision. None had any knowledge about Chinese Sign Language.

Active parental consent was obtained and only those students with written consent participated in the current study. All participants returned consent forms and received permission to participate. The Institutional Review Board of the South China Normal University (Guangzhou, China) approved this study Participants (except two of the deaf participants) were children of hearing parents.

### Materials

**Fig 1:**
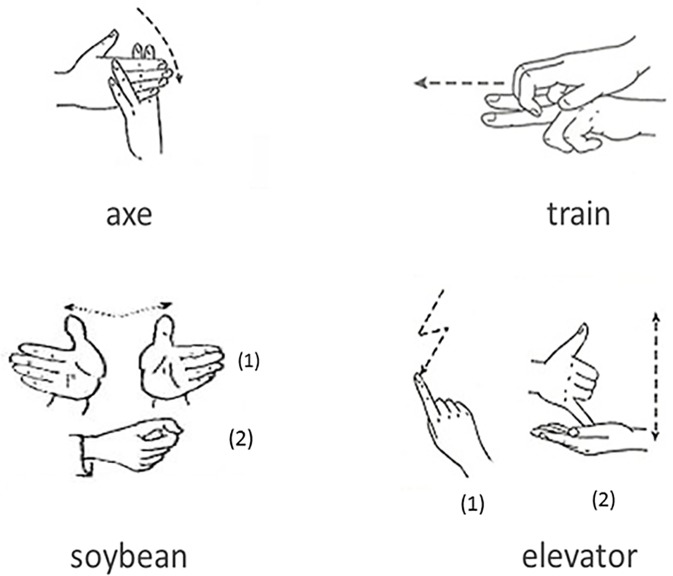
Examples of sign language for two-character Chinese words in four experimental conditions (A means ‘*axe’*, indicating the derivational word with one-sign-structure condition; B means ‘*train’*, indicating the compound word with one-sign-structure condition; C means ‘*soybean’*, indicating the derivational word with two-sign-structure condition; D means ‘*elevator’*, indicating the compound word with two-sign-structure condition).

The target word stimuli consisted of four sets of two-character Chinese polymorphemic words, each of 24 words. Two sets of words (sets 1 and 2) were derivational words with a prefix (e.g., 大豆 meaning *soybean* with 大 meaning *big* as a prefix) and/or ended with a suffix (e.g., 斧头 meaning *axe* with 头 meaning *head* as a suffix), while the other two sets of words (sets 3 and 4) were compounds with two base words (e.g., 牛奶 meaning *milk* with 牛 meaning *cow* and 奶 meaning *milk* as two individual morphemes). CSL signs for the words in the first and the third sets were formed of two sequential signs with each character formed of one sign (referred to as the DW-2 and CW-2 words respectively), and CSL signs for the words in the second and the forth sets were formed of one single sign (referred to as the DW-1 and CW-1 words respectively). For example, the sign for ‘大豆’ (soybean with signs indicating ‘big’ and ‘shape of a soyabean’; a derivational word) and ‘电梯’ (elevator with signs indicating ‘electricity’ and ‘carrying people up and down’; a compound word) were formed of two separate signs in sequential order, respectively, while the sign for ‘斧头’ (axe with a sign indicating splitting timber; a derivational word) and ‘火车’ (train with a sign indicating a car moving along the railroad; a compound word) were formed of one single sign, respectively (Fig. 1). None of the words composed of signs were assisted by facial expression.

The four sets were pretested to ensure that their comprehension would not be affected by concreteness, familiarity, or iconicity of the sign language. Ten deaf participants and ten hearing participants from the same subject pool of formal experiment who did not contribute to the test data, were asked to rate the concreteness and familiarity of each word on a 5-point *Likert* scale with 1 for least familiar or concrete and 5 for most familiar or concrete. Pictures of CSL signs for each word were presented only to the 10 hearing participants. They were asked to rate iconicity of each sign on a 5-point *Likert* scale with 1 for least iconic and 5 for most iconic. The four sets of words were balanced in subjective concreteness ratings (deaf participants: mean rating being 3.68, 3.44, 3.70, and 3.66 for DW-1, DW-2, CW-1, and CW-2 respectively, *F*(3, 92) = .71, *p* = .55; hearing participants: mean rating being 3.71, 3.51, 3.74, 3.63 for DW-1, DW-2, CW-1, and CW-2 respectively, *F*(3, 92) = .55, *p* = .65; the differences between deaf and hearing participants in each subset of words were not significant, *p* > .05), subjective familiarity ratings (deaf participants: mean rating being 3.93, 3.80, 3.95, and 3.90 for the DW-1, DW-2, CW-1, and CW-2 respectively, *F* (3, 92) = .49, *p* = .69; hearing participants: mean rating being 4.04, 3.88, 4.02, 4.00 for DW-1, DW-2, CW-1, and CW-2 respectively, *F*(3, 92) = .77, *p* = .51; the differences between deaf and hearing participants in each subset of words were not significant, *p* > .05), and iconicity of the sign language (mean iconicity rating being 3.50, 3.48, 3.66, 3.49 for DW-1, DW-2, CW-1, and CW-2 respectively, *F*(3, 92) = .75, *p* = .53).

Word frequency and strokes were also matched across the four sets of words (mean frequency being 36.95, 37.17, 36.81, and 37.00 per million for DW-1, DW-2, CW-1, and CW-2 respectively, *F*(3, 92) = .00, *p* = 1.00; mean stroke being 13.63, 13.71, 13.88, and 13.50 for DW-1, DW-2, CW-1, and CW-2 respectively, *F*(3, 92) = .04, *p* = .99). Word frequencies were obtained based on Cai and Brysbaert [31].

Ninety-six two-character non-words were added, making up 96 filler trials, 32 assigned to each block to elicit negative responses. In half of the trials, the non-words contained characters used as a prefix or suffix.

### Procedure

Participants were all tested at school in a separate room, sitting in front of a computer monitor. Following the task instructions and 24 practice trials with feedback, they completed three test blocks, each with 64 trials (i.e., 32 test trials randomly intermixed with 32 filler trials). The first two trials of each test block were filler items. Each trial started with a fixation shown in the center of the monitor for 500 ms. After a 250 ms blank screen, a word/non-word was presented. Participants were asked to judge whether it was a word or not as quickly and accurately as possible. Participants made their responses by pressing either F designated as ‘yes’ key or J designated as ‘no’ key on the keyboard. The two key-pressing responses were counterbalanced across participants. The program recorded their response time. If no response was detected within 4000 ms, the program automatically went on the next trial. The interval between trials was 250 ms. There was a one-minute break between blocks with block order counterbalanced across subjects.

Participants were allowed to proceed to the test session only if they achieved at least 90% accuracy in practice session. They could repeat practice trials until they achieved the 90% criterion.

## Results

### Deaf adolescents

**Fig 2:**
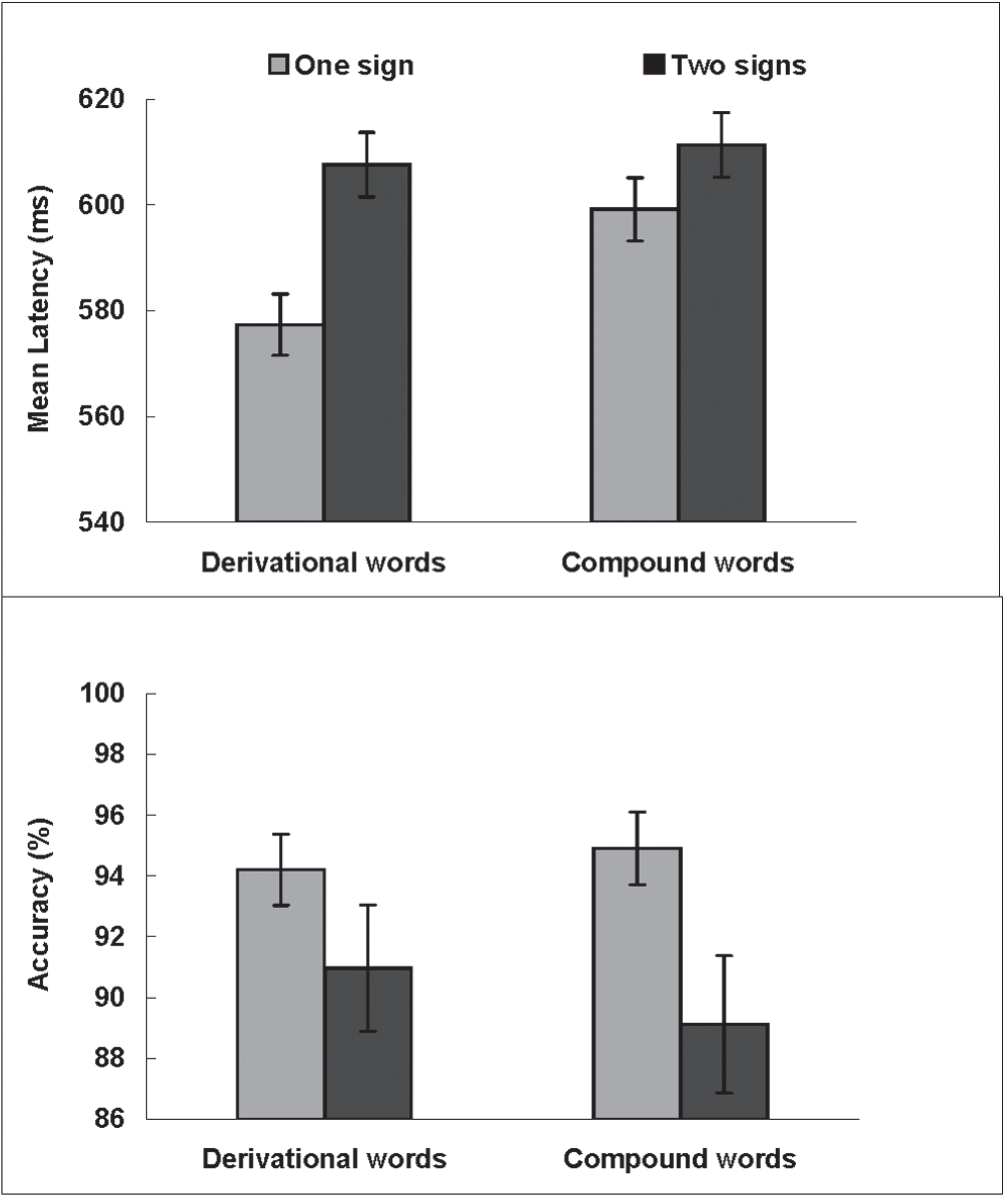
Mean latencies of correct responses (ms, upper panel) and accuracy (%, lower panel) for derivational words and compound words in each signing condition in deaf participants. Error bars show standard errors.

Reaction times and accuracy of deaf adolescents are summarized in Fig. 2. Of the 18 participants, two needed more practice to raise their accuracy rates to acceptable levels. Errors in making lexical decision occurred on 7.70% of the trials. Correct trials with reaction times lower than 300 ms and higher than 1000 ms were removed (4.68%).

Resulting latency and accuracy were submitted to a 2 (Word Type: derivational words vs. compound words) × 2 (Sign Type: two signs vs. one sign) repeated-measures ANOVA by participants and by items. In the latency analysis, there were significant main effects of Word Type (*F*_*1*_(1, 17) = 5.91, *MSE* = 496.81, *p* < .05, *η*_*p*_^*2*^ = .26; *F*_*2*_(1, 92) = 1.03, *MSE* = 3020.45, *p* = .31, *η*_*p*_^*2*^ = .01) and Sign Type (*F*_*1*_(1, 17) = 12.00, *MSE* = 675.32, *p* < .01, *η*_*p*_^*2*^ = .41; *F*_*2*_(1, 92) = 5.64, *MSE* = 3020.45, *p* <.05, *η*_*p*_^*2*^ = .06). Participants responded faster to derivational words (*M* = 592 ms) than compound words (*M* = 605 ms), and faster to words with one sign (*M* = 588 ms) than words with two signs (*M* = 609 ms). More important for the purposes of our study, we found a significant effect of sign structure on word processing, indexed by an interaction effect between Word Type and Sign Type (*F*_*1*_(1, 17) = 7.69, *MSE* = 191.28, *p* < .05, *η*_*p*_^*2*^ = .31; *F*_*2*_(1, 92) = 1.05, *MSE* = 3020.45, *p* = .30, *η*_*p*_^*2*^ = .01). When the interaction was broken down by word type, follow-up analyses showed that latencies for DW-1 were 30 ms faster on average than DW-2, *F*_*1*_(1, 17) = 13.88, *MSE* = 593.68, *p* < .01, *η*_*p*_^*2*^ = .45; *F*_*2*_ (1, 46) = 5.31, *MSE* = 3288.41, *p* < .05, *η*_*p*_^*2*^ = .10. Latencies for CW-1 were 12 ms faster on average than CW-2, *F*_*1*_(1, 17) = 4.89, *MSE* = 272.92, *p* < .05, *η*_*p*_^*2*^ = .22; *F*_*2*_(1, 46) = 1.00, *MSE* = 2752.49, *p* = .32, *η*_*p*_^*2*^ = .02. When the interaction was broken down by sign language structure, follow-up analyses showed that bilingual participants spent less time processing DW-1 (577 ms) than CW-1 (599 ms), (*F*_*1*_(1, 17) = 10.07, *MSE* = 425.21, *p* < .01, *η*_*p*_^*2*^ = .37; *F*_*2*_ (1, 46) = 3.00, *MSE* = 2102.18, *p* = .09, *η*_*p*_^*2*^ = .06). The same comparison between DW-2 and CW-2 failed to yield a significant difference (mean for derivational words = 608 ms; mean for compound word = 611 ms, (*F*_*1*_(1, 17) = .48, *MSE* = 262.88, *p* = .50, *η*_*p*_^*2*^ = .03; *F*_*2*_(1, 46) = .00, *MSE* = 3938.73, *p* = .99, *η*_*p*_^*2*^ = .00).

In the accuracy analyses, there was a significant main effect for Sign Type, *F*_*1*_(1, 17) = 14.58, *MSE* = 25.15, *p* < .001, *η*_*p*_^*2*^ = .46; *F*_*2*_(1, 92) = 4.06, *MSE* = 120.56, *p* < .05, *η*_*p*_^*2*^ = .04. The accuracy for words formed of one sign (94.6%) was higher than for words formed of separate signs (90.0%). The main effect for Word Type did not reach significance level, *F*_*1*_(1, 17) = .15, *MSE* = 39.22, *p* = .70, *η*_*p*_^*2*^ = .01; *F*_*2*_(1, 92) = .07, *MSE* = 120.56, *p* = .80, *η*_*p*_^*2*^ = .00, nor did the interaction between Word Type and Sign Type, *F*_*1*_(1, 17) = 2.58, *MSE* = 11.31, *p* = .13, *η*_*p*_^*2*^ = .13; *F*_*2*_(1, 92) = .32, *MSE* = 120.56, *p* = .57, *η*_*p*_^*2*^ = .00.

### Hearing adolescents

**Fig 3:**
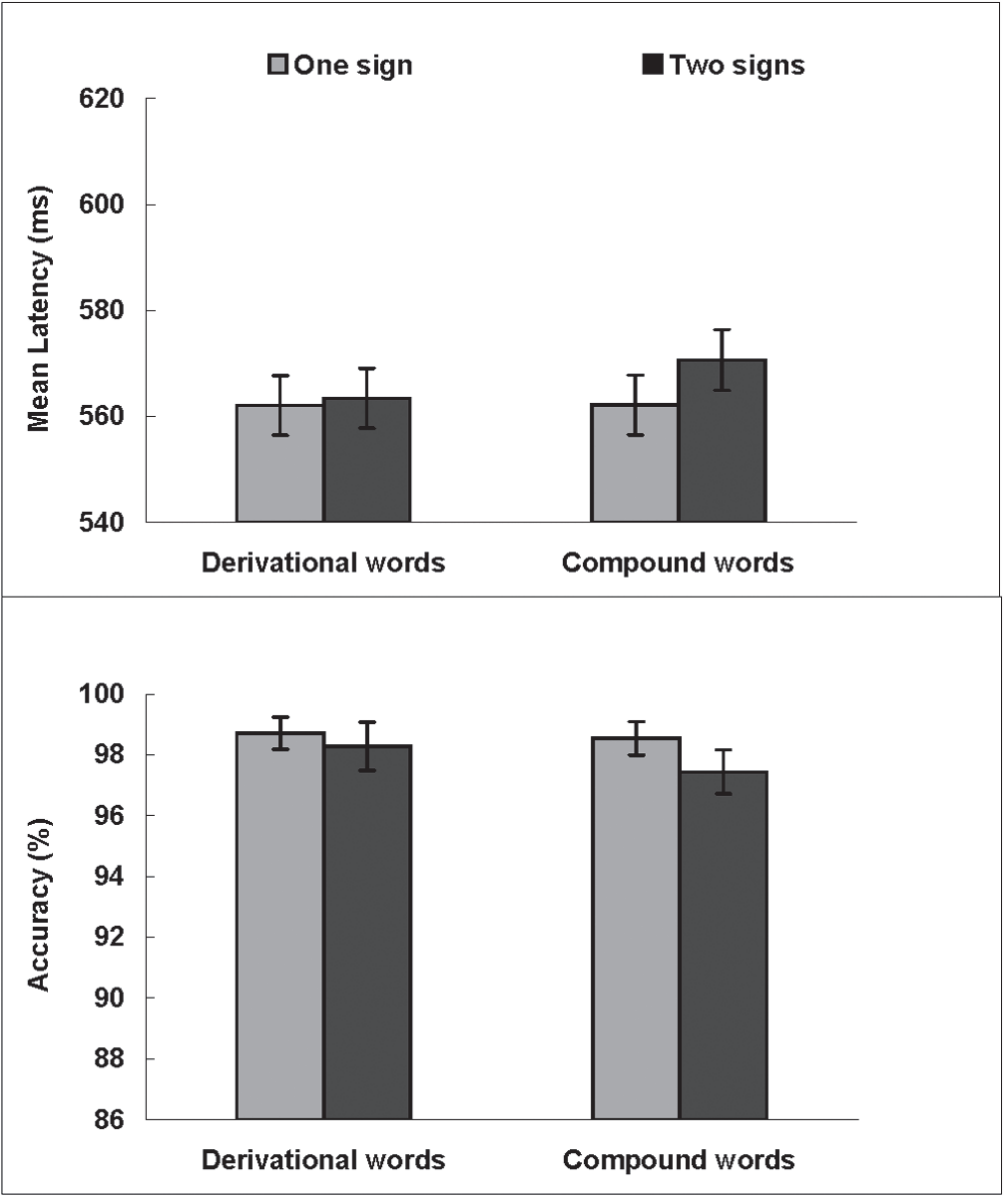
Mean latencies of correct responses (ms, upper panel) and accuracy (%, lower panel) for derivational words and compound words in each signing condition in hearing participants. Error bars show standard errors.

Fig. 3 presents reaction times and accuracy of hearing adolescents identifying target words. Of the 20 participants, no one needed more practice to raise their accuracy rates to acceptable levels. Errors in making lexical decision occurred on 1.93% of the trials. Correct trials with reaction times lower than 300 ms and higher than 1000 ms were removed (1.89%).

Resulting latency and accuracy were submitted to a 2 (Word Type: derivational words vs. compound words) × 2 (Sign Type: two signs vs. one sign) repeated measures ANOVA by participants and by items. In the latency analysis, there were no significant main effects of Word Type (*F*_*1*_(1, 19) = .86, *MSE* = 309.22, *p* = .37, *η*_*p*_^*2*^ = .04; *F*_*2*_(1, 92) = .25, *MSE* = 1601.23, *p* = .62, *η*_*p*_^*2*^ = .00), Sign Type (*F*_*1*_(1, 19) = 1.89, *MSE* = 256.08, *p* = .19, *η*_*p*_^*2*^ = .09; *F*_*2*_(1, 92) = .46, *MSE* = 1601.23, *p* = .50, *η*_*p*_^*2*^ = .01), or no significant interaction between Word Type and Sign Type (*F*_*1*_(1, 19) = .52, *MSE* = 489.21, *p* = .48, *η*_*p*_^*2*^ = .03; *F*_*2*_(1, 92) = .13, *MSE* = 1601.23, *p* = .73, *η*_*p*_^*2*^ = .00). Latencies for DW-1 (562 ms) were as fast as on average for DW-2 (563 ms), *F*_*1*_(1, 19) = .09, *MSE* = 215.63, *p* = .77, *η*_*p*_^*2*^ = .00; *F*_*2*_(1, 46) = .05, *MSE* = 1712.17, *p* = .83, *η*_*p*_^*2*^ = .00. Latencies for CW-1 (562 ms) were also as fast as on average for CW-2 (570 ms), *F*_*1*_(1, 19) = 1.36, *MSE* = 529.66, *p* = .26, *η*_*p*_^*2*^ = .07; *F*_*2*_(1, 46) = .57, *MSE* = 1490.29, *p* = .46, *η*_*p*_^*2*^ = .01. In accuracy analyses, there were also no significant main effects of Sign Type, Word Type, or interaction effect (all *p* > .05).

To investigate the delay of morphological processing in Chinese deaf adolescents and the influence of sign language structure on this delay, a repeated-measures ANOVA was carried out with Word Type (compound words vs. derivational words) and Sign Type (two signs vs. one sign) as within-subject factors and Group (deaf adolescents vs. hearing adolescents) as a between-subject factor. This analyses showed significant main effects of Word Type, Sign type, and Group (all *p* < .05), as well as the two-way interaction between Sign type and Group (*F*_*1*_ (1, 36) = 5.54, *MSE* = 454.05, *p* < .05, *η*_*p*_^*2*^ = .13; *F*_*2*_ (1, 92) = 2.26, *MSE* = 2372.53, *p* = .14, *η*_*p*_^*2*^ = .02). Deaf adolescents responded slower to words with two signs than hearing peers (609 ms vs. 567 ms, *F*_*1*_ (1, 36) = 7.64, *MSE* = 4459.99, *p* <.01, *η*_*p*_^*2*^ = .18; *F*_*2*_ (1, 94) = 18.48, *MSE* = 2868.83, *p* < .001, *η*_*p*_^*2*^ = .16), while they were comparable in responding to words with one sign (588 ms vs. 562 ms, *F*_*1*_ (1, 36) = 2.46, *MSE* = 5249.34, *p* = .13, *η*_*p*_^*2*^ = .06; *F*_*2*_ (1, 94) = 9.29, *MSE* = 1727.57, *p* < .05, *η*_*p*_^*2*^ = .09). Most importantly, there was a significant three-way interaction between Word Type, Sign type, and Group (*F*_*1*_ (1, 36) = 4.31, *MSE* = 348.52, *p* < .05, *η*_*p*_^*2*^ = .11; *F*_*2*_ (1, 92) = 1.04, *MSE* = 2372.53, *p* = .31, *η*_*p*_^*2*^ = .01). Deaf adolescents were much slower than the hearing adolescents when responding to DW-2 (608 ms vs. 563 ms; *F*_*1*_ (1, 36) = 7.08, *MSE* = 2609.02, *p* < .05, *η*_*p*_^*2*^ = .16; *F*_*2*_ (1, 46) = 9.28, *MSE* = 3300.04, *p* < .01, *η*_*p*_^*2*^ = .17), while there was no significant difference between groups when responding to DW-1 (577 ms vs. 562 ms; *F*_*1*_ (1, 36) = .83, *MSE* = 2655.13, *p* = .37, *η*_*p*_^*2*^ = .02; *F*_*2*_ (1, 46) = 1.59, *MSE* = 1700.55, *p* = .21, *η*_*p*_^*2*^ = .21). Similarly, deaf adolescents responded slower to CW-2 (611 ms) than the hearing adolescents (571 ms; *F*_*1*_ (1, 36) = 7.01, *MSE* = 2235.19, *p <* .05, *η*_*p*_^*2*^ = .16; *F*_*2*_(1, 46) = 8.89, *MSE* = 2549.77, *p* < .01, *η*_*p*_^*2*^ = .16). However, they also responded slower to CW-1 (599 ms) than the hearing adolescents (562 ms; *F*_*1*_ (1, 36) = 4.38, *MSE* = 2956.32, *p <* .05, *η*_*p*_^*2*^ = .11; *F*_*2*_ (1, 46) = 9.55, *MSE* = 1693.02, *p* < .01, *η*_*p*_^*2*^ = .17). The other interactions were not significant (*p* > .05). The results showed that the deaf adolescents were slower in reading DW-2, CW-1, and CW-2 than hearing adolescents, but not DW-1. Specifically, it seems that deaf adolescents had the greatest difficulties in processing DW-2 (45 ms), then in CW-2 (40 ms), and lastly in CW-1 (37 ms).

### Discussion

The results showed that the processing of a word was relatively slower and less accurate if its CSL was formed of two signs and such effect was stronger in derivational words. These results suggested that the sign language was activated during written language comprehension, and extended that the structure of sign language would have an impact on written language processing with its effect varying as a function of word type. Our results suggested the existence of bottom-up influence of sign language structure on Chinese written words processing. Due to the difficulty in finding a set of two-character monomorphemic words which have no corresponding signs but are matched with the test words in words frequencies, stroke numbers, concreteness, familiarity, and iconicity, the current study did not include a set of control words. Anyway, the results of current study did provide evidence for the effect of sign language structure on print morphological processing with a possible processing disadvantage in words with two signs and a possible processing advantage in words with one sign. Moreover, the discrepancy in processing between one-sign and two-sign words was greater in derivational words than in compound words.

The results also showed that Chinese deaf readers responded faster to derivational words than to compound words. Though it was different from the finding of van Hoogmoed et al. [6] that Dutch deaf readers responded faster to compound words than derivational words, it did suggest that Chinese deaf adolescents acquire print morphological awareness. Moreover, the advantage in derivational words was greater in the one-sign-structure condition than in the two-sign-structure condition. We discussed this point in detail in the section of general discussion.

Additionally, it showed that the response latencies were comparable between derivational and compound words in hearing adolescents, consistent with the finding in Experiment 2 of van Hoogmoed et al. [6]. Moreover, the comparison of deaf and hearing adolescents showed that the structure of sign language did affect the delay processing of written language in deaf adolescents, and its effect varied as a function of separation or not of the sign structure and the written word type (i.e., compound or derivation words). Previous studies have consistently found that there was a lower accuracy in affix split decision task in deaf signers, suggesting a delay in the development of their print morphological awareness [19]. Moreover, though reading level had been matched between deaf and hearing readers, poor print morphological word analysis was still found in deaf readers, and their poor print morphological awareness further resulted in poor literacy skills [19]. We ensured that all test words (i.e., derivational and compound words) were familiar to both deaf and hearing adolescents, however, delayed processing of DW-2, CW-1, and CW-2 were found in deaf adolescents with the largest delay in DW-2 but no delay in DW-1, suggesting the structure of sign language affect the delay pattern of print morphological processing.

## General Discussion

While many recent studies have examined the delayed development of written word comprehension in deaf people, the present study examined the effect of sign language structure on complex word reading and on the delayed print morphological processing in Chinese deaf adolescents. We found that Chinese deaf adolescents responded faster to derivational words than compounds in the one-sign-structure condition, but showed comparable performance in the two-sign-structure condition. And the latencies for words with one-sign structure were shorter than those with two-sign structure in both derivational and compound words. These results provide strong evidence that the structure of sign language affects written word processing, suggesting a bottom-up structure effect of sign language on Chinese word reading. Additionally, the difference between derivational words and compound words in one-sign-structure condition indicates that Chinese deaf adolescents have print morphological awareness. The results also showed that delayed written word reading in DW-2, CW-1, and CW-2, but not in DW-1, with the delay being maximum in DW-2, medium in CW-2, and minimum in CW-1, suggesting that the structure of sign language has an impact on the processing of Chinese written words.

The present study focusing on the effect of sign language on written word processing in sign-print bilinguals, contribute to a burgeoning literature for the co-activation of both languages in bimodal bilinguals. Previous studies have showed that bimodal bilinguals can produce lexical items from both languages at the same time [32]. Similarly, there is evidence showing that bimodal bilinguals look more at competing items that overlap with the target in the phonology of American Sign Language than at phonologically unrelated items, and more at competing items relative to monolinguals [14]. Basic language constructs considered essential for reading success include phonological and phonemic awareness and morphology [33]. For the deaf bilinguals, these language constructs also include the phonological and morphological properties of sign language, such as motion and posture found in previous studies (i.e., phonology) and the separation-or-not structure of sign language (i.e., morphology) found in present study. Thus, combined with previous research, the effect of sign language on spoken/written language is not confined to their common semantic representations but is also present in the phonological and morphological representation of sign language, namely the bottom-up effect.

An interesting finding is that the effect of sign language structure varies across the two types of Chinese words (i.e., derivational and compound words). There was a significant difference between derivational and compound words when they were formed of one sign, but their difference disappeared when formed of two signs. Moreover, latencies for words with one sign were shorter than those with two signs in both types of words. Firstly, previous studies appeared to support a lexeme-based rather than morpheme-based model of morphological representation [34–35]. When the Chinese two-character words are formed of one sign, they are represented as one lexeme in sign language. When they are formed of two signs, they are represented separately and thus need extra time to integrate the two sequential separate signs into one. That may be why words with one sign were responded to faster than those with two signs. Additionally, as Chinese derivational words could be discriminated through its relatively fixed structure, and given the relatively small number of affixes [36], they would be represented as one lexeme in mental lexicon, while compound words are judged by understanding the internal syntactic rules and thus would be represented with separate lexemes in mental lexicon. Therefore, it is understandable why Chinese derivational words were responded to faster than its compound words.

Secondly, when taking sign language into consideration, the response time of derivational words did not differ from that of compound words in two-sign condition, which may suggest that the two-sign structure delays the processing of derivational words, resulting in response times in DW-2 comparable with CW-2. Additionally, the shorter response time found in CW-1 relative to CW-2, may suggest that one-sign structure strengthens the closer connections of two characters in compound words. That is, the structure of sign language modulates the processing of Chinese written words. Moreover, as derivational words depend on structure, it is possible that the structure of sign language has a stronger effect on derivational word making its discrimination time shorter in one-sign condition and longer in two-sign condition. Such a speculation, while interesting, will require more evidence. Assuming an interactive model of word recognition [37], our result is a possible indication that the spread of activation across the lexicons of Chinese written words and linguistic signs is more specific than we expected.

The present study also shows that the structure of sign language influences the delay pattern of written word reading in Chinese deaf adolescents with delay found in DW-2, CW-1, and CW-2, but not DW-1. That is, DW-1 spares the delay while DW-2 suffers the severest delay. Our results are consistent with previous studies which showed that the median reading comprehension skills for deaf 17-year-olds were at a fourth-grade (9-year-old) level [1], suggesting severe delay in deaf adolescents. However, unlike previous studies, we further showed that sign language would have positive as well as negative effects on the development of Chinese word reading for deaf adolescents. On the one hand, the spared delay in DW-1 may be because the lexemes of DW-1 in written Chinese and sign language are consistent in that both of them are represented as one coherent lexeme in each language. Thus, DW-1 would be easier for deaf adolescents to learn and could achieve the same processing level as their hearing peers before or during adolescent period. However, among the rest three types of words (i.e., DW-2, CW-1, and CW-2), DW-2 showed the severest delay, which may be due to the fact that the lexemes of DW-2 in written Chinese and sign language are inconsistent in that DW-2 in written Chinese was represented as one lexeme but in sign language as two separate ‘lexemes’. Thus, DW-2 would be harder for deaf adolescents to learn and could not achieve the same processing level as their hearing peers.

Recent studies have demonstrated crossover effects of print morphological awareness on biliteracy acquisition across alphabets [38]. Our findings further showed the crossover effects of sign language structure exerting on Chinese written word processing and its delayed processing relative to hearing peers, and provide insight into the mechanisms about how co-activation of unimodal languages affects language processing and delayed processing in sign-print bilinguals.

This study contributes to relevant literature because the effect of sign language structure had never been explored in relation to complex word processing. The study has limitations to be addressed in future research. Although we provide evidence that the structure of sign language affects complex word reading and its delayed processing in deaf adolescent readers, future studies should confirm this conclusion by involving a control group with equivalent reading age for comparison. Specifically, it has been found that deaf adolescents are as accurate as hearing children of the same reading age, though invoking different strategies [39]. The present study used hearing peers of the same age but not the same reading age to reveal the delay processing of complex word, and found the effect of sign language structure on this delay. To reveal the specific effect of sign language structure on the lag of written reading skills, a control group with equivalent reading age for comparison should be added in future study. Additionaly, it is possible that deaf adolescents are not only disadvantaged in reading because of schooling and language lag problems but because they are bilinguals themselves. The present study pointed to a link between CSL structure and a delayed comprehension, but it is necessary to involve a comparision control group of hearing bilinguals or equivalent to see if hearing adolescents have a lag when they are too bilingual in future research.

Obviously, our findings have two practical implications. On the one hand, we argue that one of the effective educational interventions that can be implemented easily and not costly to improve deaf people’s written language acquisition is to strengthen their learning of sign language, especially facilitating the integration of multiple separate signs of a word into one. On the other hand, the bottom-up effect of sign language structure suggests that the easier and simpler the structure of sign language is, the faster the written language is processed. Thus, for a better understanding of sign language as well as their equivalent written language, a coherent structure avoiding separating a word into different independent signs should be recommended in designing the sign language.
